# The co-stimulatory molecule B7-H3 promotes the epithelial-mesenchymal transition in colorectal cancer

**DOI:** 10.18632/oncotarget.9035

**Published:** 2016-04-27

**Authors:** Bo Jiang, Ting Zhang, Fen Liu, Zhangzhang Sun, Hanping Shi, Dong Hua, Chen Yang

**Affiliations:** ^1^ Department of Medical Oncology, Beijing Institute of Translational Medicine, Chinese Academy of Sciences/Cancer Center, Aviation General Hospital, Beijing, China; ^2^ Institute of Cancer, Affiliated Hospital of Jiangnan University, Wuxi, Jiangsu, China; ^3^ Department of Medical Oncology, Affiliated Hospital of Jiangnan University, Wuxi, Jiangsu, China; ^4^ Department of Nuclear-Medicine, Suzhou Hospital Affiliated to Nanjing Medical University, Suzhou, Jiangsu, China

**Keywords:** B7-H3, colorectal cancer, epithelial-mesenchymal transition, co-stimulatory molecule, metastasis, Immunology and Microbiology Section, Immune response, Immunity

## Abstract

B7-H3, first recognized as a co-stimulatory molecule, is abnormally expressed in cancer tissues and is associated with cancer metastasis and a poor prognosis. However, as an initial event of metastasis, the relationship between the Epithelial–Mesenchymal Transition (EMT) in cancer cells and B7-H3 has still not been investigated. In this study, we first analyzed B7-H3 expression by immunohistochemistry in colorectal cancer tissues. B7-H3 was expressed in the cancer cell membrane and was associated with the T stage of colorectal cancer; it also showed a positive correlation with MMP2 and MMP9 expression in cancer tissues. Over-expression of B7-H3 in SW480 cells allowed cancer cells to invade and metastasize more than the control cells, whereas invasion and metastasis capabilities were decreased after B7-H3 was knocked down in Caco-2 cells. We further showed that B7-H3 down-regulated the expression of E-cadherin and β-catenin and up-regulated N-cadherin and Vimentin expression, implying that B7-H3 promoted the EMT in colorectal cancer cells. We also checked another character of the EMT, the stemness of cancer cells. CD133, CD44 and Oct4 were significantly elevated after the SW480 cells were transfected with B7-H3 and reduced in Caco-2 cells after B7-H3 was inhibited. In subsequent studies, we proved that B7-H3 upregulated the expression of Smad1 via PI3K-Akt. In conclusion, B7-H3 promotes the EMT in colorectal cancer cells by activating the PI3K-Akt pathway and upregulating the expression of Smad1.

## INTRODUCTION

Colorectal cancer is the third most diagnosed cause of cancer death in the world [1]. Metastases are frequently observed in colorectal cancer. During the whole process of these diseases, approximately 50% of patients have liver, lung or other organ metastases, and 20% patients already have liver metastases when their colorectal cancer is diagnosed [2].

The process of cancer metastasis can be divided into six sequential steps, including isolation from the cancer tissue, intravasation, survival in the circulatory system, extravasation, homing, and colonization. In the initial step of cancer metastasis, the invading cancer cells lose cell-cell adhesion, increase their motility, and secrete matrix metalloproteinases (MMPs) to dissolve the basal membrane [3]. At the same time, the cancer cells show high plasticity, including morphological and phenotypical conversions, named the epithelial to mesenchymal transition (EMT) [4, 5]. The mesenchymal to epithelial transition (MET) describes the reverse process. The cancer cells undergoing EMT or MET are influenced by their microenvironment [6], hypoxia [7], cytokines [8], etc.

The association between inflammatory bowel disease (IBD) and colorectal cancer (CRC) has been recognized since 1925 [9]. Inflammatory reactions, inflammatory factors (TGF-β, IL-6, etc.) and immune cells are associated with the formation of malignant colon tumors [10]. T cells play a central role in IBD and cancer growth and progression [11, 12] because they help B cells produce antibodies, secrete cytokines and chemokines, and kill virus-infected cells and cancer cells. The proliferation and activity of T cells, including both CD4^+^ T cells and CD8^+^ T cells, are regulated by multiple receptor-ligand interactions. At least two signals are required for T cell activity, one is the major histocompatibility complex (MHC), and the second is a co-stimulatory signal that is activated by co-stimulatory molecules [13, 14].

The B7-CD28 superfamily has been shown to be important for regulating the responses of previously activated T cells, and they function as co-stimulatory or co-inhibitory molecules. The B7 superfamily includes eight members: B7-1, B7-2, B7-DC, B7-H1, B7-H2, B7-H3, B7-H4 and B7-H6 [15]. All B7 superfamily [16] members are transmembrane proteins that contain an extracellular domain, an intracellular domain and a transmembrane domain. B7-H3 is encoded by the CD276 gene, including two main isoforms, named 4Ig-B7-H3 and 2Ig-B7-H3, which were first identified in 2001 [17, 18]. 4Ig-B7-H3 contains two immunoglobulin-like V and C domains (VC domains) and is dominantly expressed in *Homo sapiens*. However, the mouse B7-H3 transcript only contains a single VC domain (2Ig-B7-H3) [19].

Similar to other B7 molecules, B7-H3 was first recognized as an immune molecule that was expressed in antigen-presenting cells (APCs) or macrophages to regulate the function of T cells as a second signal molecule. Recently, B7-H3 has been reported to promote cancer metastasis in patients with higher levels of B7-H3 expression, including gastric cancer [20], prostate cancer [21], pancreatic cancer [22], glioma [23] and colorectal cancer [24]. It was also proved that B7-H3 promoted MMP expression [25] and induced drug resistance in cancer cells [26, 27]. Therefore, the non-immunological efficiency of B7-H3 has gradually been accepted by clinical researchers and immunologists. Based on our previous results, we had proved that over-expressed B7-H3 could active the Jak2-STAT3 signaling pathway to augment the anti-apoptotic activity, migration and invasion of cancer cells [28].

Colorectal cancer has a close relationship with chronic inflammation and the EMT [29]. B7-H3 is an important mediator of the chronic inflammation process and promotes colorectal cancer metastasis, implying that B7-H3 molecules might play a key role in the malignant transformation and the EMT in colorectal epithelial cells. In this study, we hypothesized that B7-H3 induces the EMT in colorectal cancer and attempted to discover the mechanism by which B7-H3 promotes cancer cell invasion and metastasis.

## RESULTS

### B7-H3 was co-expressed with metalloproteinases in colorectal cancer

**Figure 1:**
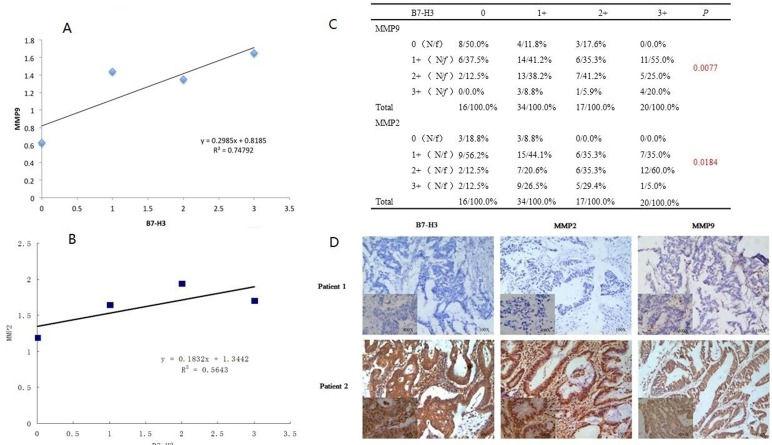
**A.** Linear regression analysis was used to investigate the relationship between B7-H3 and MMP9. **B.** Linear regression analysis was used to investigate the relationship between B7-H3 and MMP2. **C.** The cohort was divided into four groups according to the B7-H3 immunohistochemical results. Based on the expression of MMP2 or MMP9 by immunohistochemical staining, MMP2 or MMP9 expression was assigned scores from 0 to 3, where 0 is negative, and 1+, 2+ or 3+ are positive staining. The mean MMP2 and MMP9 expression levels were calculated using following formula: $Meanscore=\frac{\sum^{\text{​}}(n^{*}score)}{N}$ n is the number of each MMP2 or MMP9 subgroup, N is the individual amount per group. **D.** The immunohistochemical staining results of B7-H3, MMP2 and MMP9 in the CRC tissues. Patient 1 displayed negative staining, and patient 2 displayed positive staining.

We randomly chose 87 colorectal cancer tissue samples to analyze the expression levels of B7-H3, MMP2 and MMP9 by immunohistochemistry (Figure 1D). All patients were divided into four groups according to the expression level of B7-H3. There were no significant differences in the patients’ gender, age and regional lymph node metastasis among the four groups, but B7-H3 over-expression increased the T stage of patients (*p* < 0.001) (Table 1). These results indicated that the expression of B7-H3 in the patients’ colorectal cancer tumor tissues was obviously associated with the depth of cancer invasion but did not have a close relationship with regional lymph node metastasis.

**Table 1:** The colon cancer tissue samples from 87 patients were divided into four groups based on the results of the B7-H3 immunohistochemical analysis

B7-H3		0	1+	2+	3+	*P*
Gender	Male	11	17	4	10	0.0745
	Female	5	17	13	10	
Age		62.2±13.7	60.7±13.3	60.2±13.4	60±13.8	0.9787
TNM stage*	I	9	13	6	2	0.1544
	II	3	10	6	10	
	III	4	11	5	8	
T	T1~T2	10	15	6	2	**0.0101**
	T3~T4	6	19	11	18	
N	N0	12	23	12	12	0.2425
	N1	4	5	2	7	
	N2	0	6	3	1	

“0” means negative, “1+” means faint, “2+” means moderate, and “3+” means strong.

* All samples were colon cancer surgical specimens from patients without distant metastasis.

As shown in Figure 1C, the patients’ tumor samples were divided into four groups based on their B7-H3 scores. Both the intensity and frequency of the MMP2 and MMP9 staining were increasingly correlated with those of B7-H3 in the CRC tissues.

To accurately calculate the relationship between the expression of MMP2 or MMP9 and B7-H3, we used a linear regression model to further analyze the data. The tumor sample cohort was divided into four subgroups, 0 to 3. Based on the expression of MMP2 or MMP9 by immunohistochemical staining, MMP2 and MMP9 expression was assigned scores from 0 to 3, where 0 is negative staining. and +1, +2, or +3 are positive staining. The mean values of MMP2 and MMP9 expression were calculated using the following formula: $Meanscore=\frac{\sum^{\text{​}}(n^{*}score)}{N}$, where n is the number of each MMP2 or MMP9 subgroup and N is the Total number of each B7-H3 group.

Based on the above results, we then analyzed these data using the linear regression model and proved that the expression of MMP9 was positively correlated with B7-H3 expression, y = 0.2987x+0.8193, R^2^ = 0.74766 (Figure 1A). Moreover, B7-H3 had a positive relationship with MMP2 expression in colorectal cancer, y = 0.1843x+1.3442 R^2^ = 0.5643 (Figure 1B).

### B7-H3 enhanced colon cancer cell invasion and migration *in vitro*

In this study, we constructed four stably transfected colon cancer cell lines using SW480 and Caco-2 cells. SW480-B7-H3 cells were stably transfected with pIRES2-B7-H3-EGFP to up-regulate B7-H3 expression in SW480 cells, and CaCo-2-shB7-H3 cells were stably transfected with the B7-H3 shRNA to knockdown the expression of B7-H3. At the same time, the cells transfected with pIRES2-EGFP were used as negative controls (SW480-NC and CaCo-2-NC).

**Figure 2:**
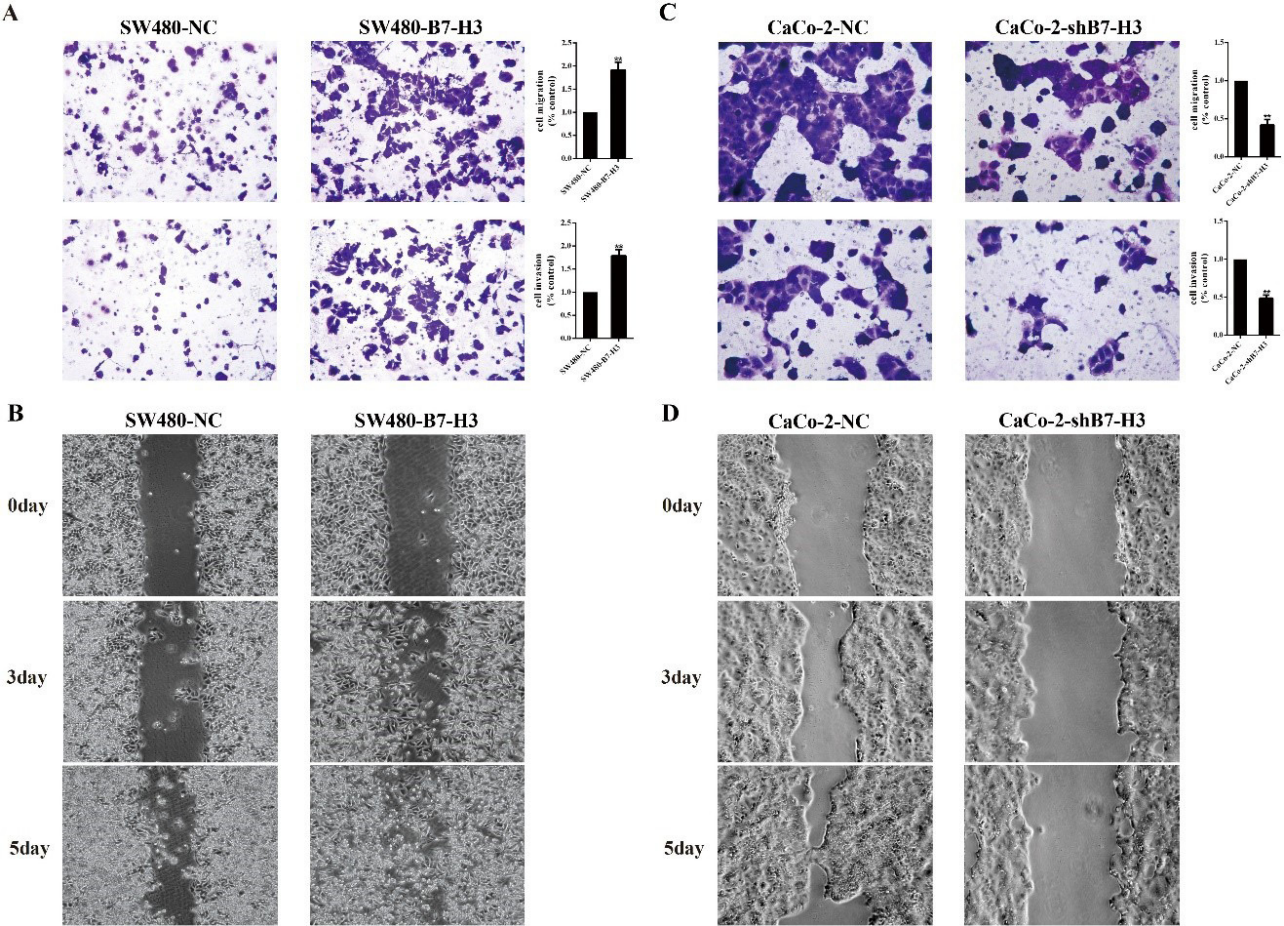
A Upper panel, evaluation of SW480-NC or SW480-B7-H3 cell migration using Transwells (without Matrigel) **A.** Lower panel, evaluation of SW480-NC or SW480-B7-H3 cell invasion using Transwells (with Matrigel). After a 24 (migration) or 48 h (invasion incubation at 37°C, the pierced cells were fixed with 4% paraformaldehyde, stained with 0.01% crystal violet, and then quantified on a microplate reader. **C.** Upper and lower panels, evaluation of CaCo2-NC or CaCo2-shB7-H3 cell migration and invasion using the same methods as for the SW480 pairs. **B.**, **D.** Wound healing assay to evaluate the migration of SW480-NC *vs* SW480-B7-H3 cells and CaCo-2-NC *vs* CaCo-shB7-H3 cells at 3 and 5 days.

We used Transwell inserts with an 8 μm thick transparent polyester membrane (Corning, New York, NY, United States) to evaluate the cells’ invasion (with Matrigel) and migration (without Matrigel) capacities. The results showed that the SW480-B7-H3 cells, which over-expressed B7-H3, migrated and invaded more efficiently than the SW480-NC cells (1.9±0.2 :1 and 1.8±0.15:1, respectively Figure 2A). We performed a similar study on the Caco-2-NC and Caco-2-shB7-H3 cells, in Caco-2-shB7-H3 cells, B7-H3 was down-regulated and showed that both the invasion and migration capability were significantly inhibited by more than 50 percent (Figure 2B).

A wound healing assay was also used to observe the differences between the SW480-NC and SW480-B7-H3 cells and Caco-2-NC and Caco-2-shB7-H3 cells. Figure 2C showed that the colorectal cancer cells with higher levels of B7-H3 exhibited a significant increase of the migration at 3 and 5 d compared with the corresponding control samples.

### B7-H3 down-regulated E-cadherin expression and up-regulated N-cadherin expression

**Figure 3:**
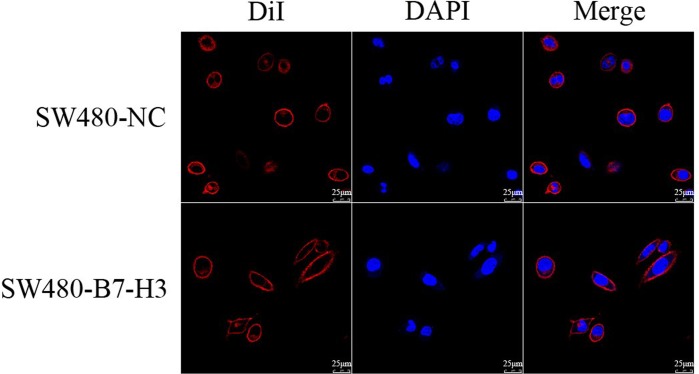
Confocal Imaging of the SW480-NC and SW480-B7-H3 cells The SW480 and SW480-B7-H3 cells were stained with DiI to label the cell membrane and with DAPI to label the nucleus. The cell shape changed from cobblestone-like to long spindle-shaped cells after the SW480 cells were transfected with B7-H3.

The loss of cell polarity is a fundamental event during the EMT. Figure 3 showed that the shape of the SW480 cells had significantly changed from a cobblestone-like shape to a long spindle shape; this transformation suggested that the SW480 cells had underwent the epithelial to mesenchymal transition.

**Figure 4:**
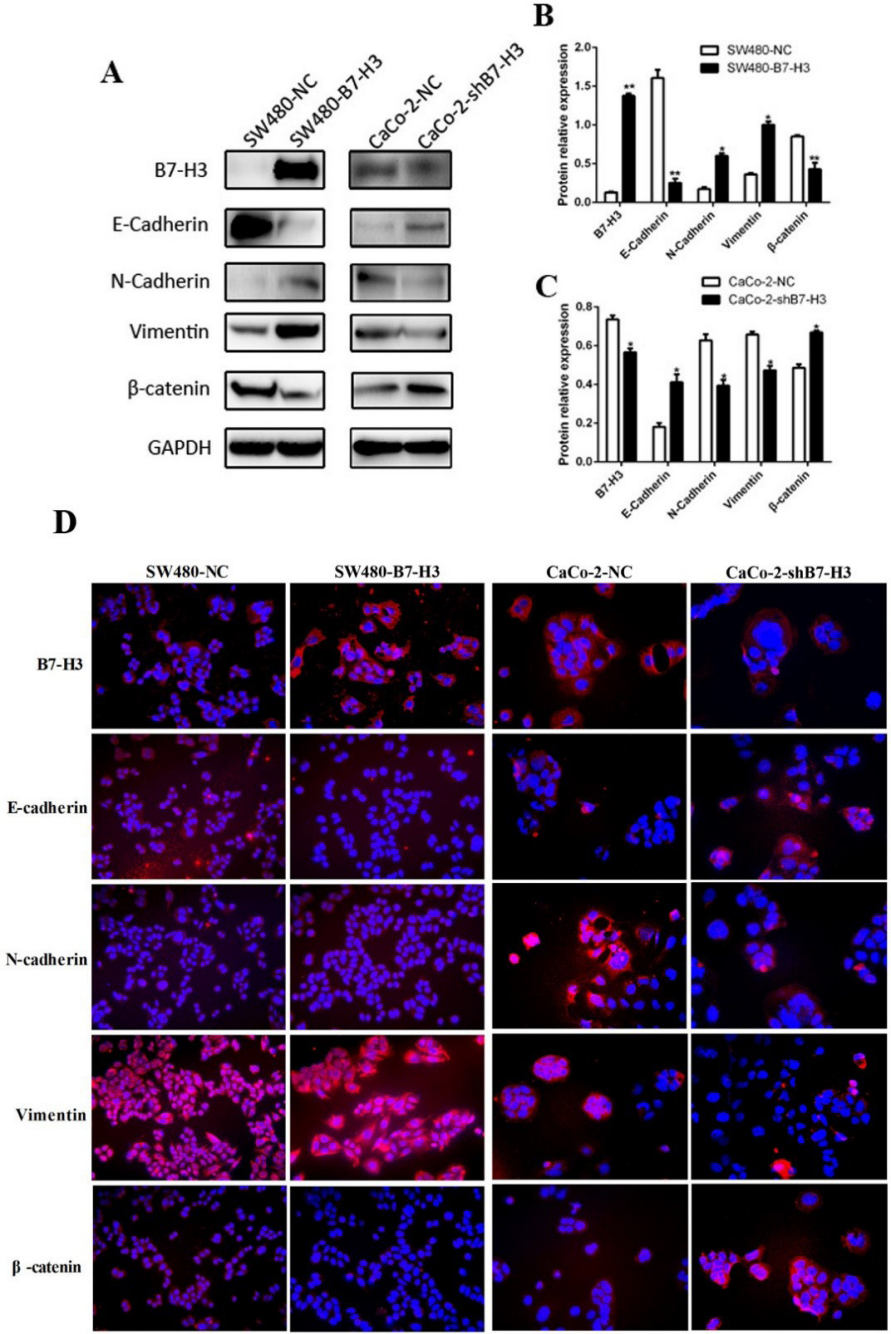
**A.** The expression of E-Cadherin, N-Cadherin, Vimentin and β-catenin in the SW480-NC, SW480-B7-H3, CaCo2-NC and CaCo2-shB7-H3 cells was detected by western blotting. **B.**, **C.** The density of each band in A was obtained with ImageJ 1.48. The density of the E-Cadherin, N-Cadherin, Vimentin and β-catenin bands in the SW480-NC and CaCo2-NC lysates were compared with those from other homologous cell lines. Differences were considered to be statistically significant when the *p* values were <0.05. **D.** Images of immunofluorescence staining of the SW480-NC, SW480-B7-H3, CaCo2-NC and CaCo2-shB7-H3 that were incubated with E-Cadherin, N-Cadherin, Vimentin, or β-catenin monoclonal antibodies and PE-labeled secondary antibodies.

To obtain stronger evidence to support this hypothesis, we analyzed the expression of E-Cadherin, N-Cadherin, Vimentin and β-catenin by western blotting. Figure 4A showed that E-Cadherin and β-catenin were down-regulated by B7-H3 overexpression. In contrast, the expression of the mesenchymal markers N-Cadherin and Vimentin was increased compared with the SW480-NC. The results proved that the B7-H3-overexpressing colorectal cancer cells had lost their epithelial characteristics and had more mesenchymal characteristics. The same results were also shown in another experiment. The CaCo-2-shB7-H3 cells in which the B7-H3 expression level had been knocked down tended to exhibit more epithelial characteristics than mesenchymal characteristics.

To intuitively describe these changes, we captured several images of the immunofluorescently stained cancer cells. All cells were stained with B7-H3, E-Cadherin, N-Cadherin, Vimentin and β-catenin monoclonal antibodies, and then incubated with a PE-labeled secondare antibody. As shown in Figure 4D, the SW480-B7-H3 cells displayed a stronger red fluorescence in the cell membrane when the cells were stained with B7-H3, N-Cadherin and Vimentin antibodies, and the display was a weaker red fluorescence when the cells were stained with E-Cadherin and β-catenin antibodies compared with the SW480-NC cells. The results were similar to the western blot results. We also used the CaCo-2-shB7-H3 cells in a complementary study and showed that the red fluorescence was stronger (E-Cadherin and β-catenin) or weaker (N-Cadherin and Vimentin) than their counterpart CaCo-2-NC cells.

Regardless of whether the expression of B7-H3 was upregulated in the SW480-B7-H3 cell line or downregulated in Caco-2-shB7-H3 cell line and whether WB or immunofluorescence was used for the analysis, B7-H3 expression induced the cancer cells to lose their epithelial markers and exhibited mesenchymal characteristics.

### B7-H3 promoted the expression of CD133, CD44 and Oct4

**Figure 5:**
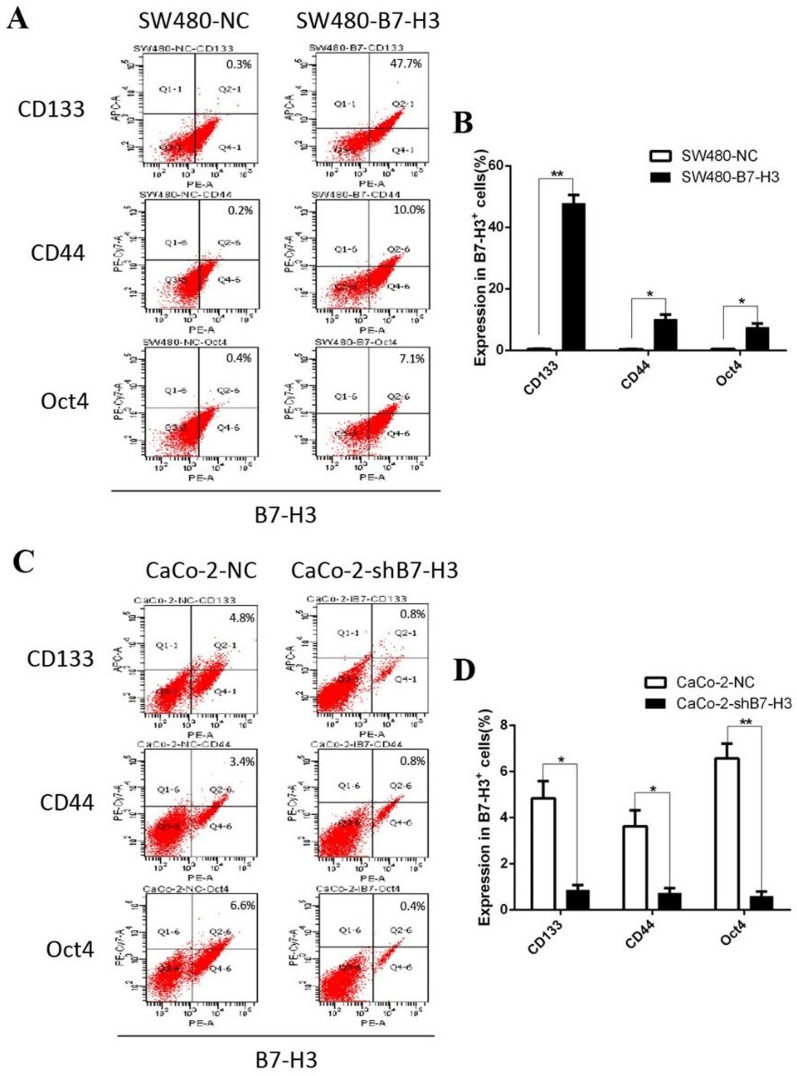
**A.** The levels of the cancer stem cell markers CD133, CD44 and Oct4 were measured in SW480-NC and SW480-B7-H3 cells by flow cytometry (FCM). **B.** Column chart and Student's T-test based on the FCM data in A. When the *p* values were < 0.05, the differences were considered statistically significant. **C.** and **D.** The same methods were used in the CaCo-2-NC and CaCo-2-shB7-H3 cells. **E.** The CD133, CD44 and Oct4 levels were measured in wild type colorectal cancer cells by FCM. **F.**, **G.**, **H.** The wild type colorectal cancer cell lines were divided into the B7-H3+ and B7-H3- subgroups according to their expression levels of B7-H3 on the cell membrane.

During the EMT, cancer cells displayed more stem cell characteristics to promote self-renewal and decrease their sensitivity to chemotherapeutic drugs. CD133, CD44 and Oct4 are commonly used as cell membrane biomarkers to identify cancer stem cell. We measured CD133, CD44 and Oct4 expression by flow cytometry and investigated the fluorescent intensity of B7-H3 staining in colorectal cancer cells. After the SW480 cells were transfected with B7-H3, the of CD133^+^ cells increased from 0.3% to 47.7%, the percentage of CD44^+^ cells increased from 0.2% to 10%, and the percentage of Oct4^+^ cells was also signaificantly increased from 0.4% to 7.1% (Figure 5A, 5B). In contrast, in the Caco-2-shB7-H3 cells in which the expression of B7-H3 was knocked down, the percentages of the CD133^+^ cells, CD44^+^ cells and Oct4^+^ cells decreased from 4.8% to 0.8%, 3.4% to 0.8% and 6.6% to 0.4%, respectively (Figure 5C, 5D). These results supported a new hypothesis that colorectal cancer cells with higher B7-H3 levels expressed more cancer stem cell markers.

### B7-H3 upregulated Smad1 expression *via* PI3K-Akt pathway

**Figure 6:**
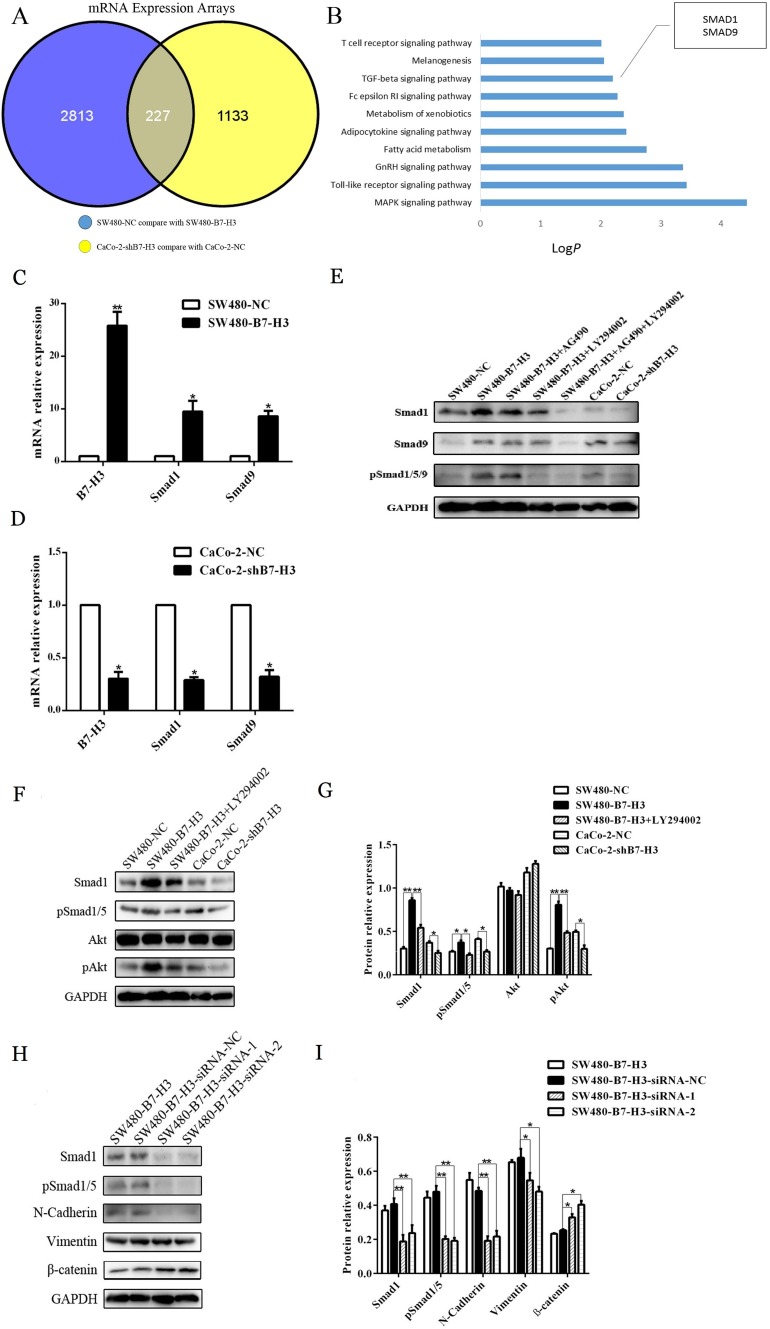
**A.** Agilent human 8×60 transcription microarray chips were used to scan the mRNA levels. The SW480-B7-H3 cells have 2,813 genes with higher expression levels than the SW480-NC cells (blue round). The Caco-2-NC cells have 1,133 genes with higher expression levels than the Caco-shB7-H3 cells (yellow round), and the intersection of these two assemblages is 227 genes. **B.** KEGG Pathway enrichment analyses were used to cluster more than 227 genes; the expression of the Smad1 and Smad9 genes in the TGF-β signaling pathway were significantly higher in the B7-H3-overexpressing cells than the controls. **C.**, **D.** The changes in the expression of Smad1 and Smad9 were verified by Real-time Quantitative PCR. The Smad1 and Smad9 mRNA levels in the SW480-B7-H3 cells are higher than those in the SW480-NC cells (Figure 6C). The Smad1 and Smad9 mRNA expression levels in the Caco-2-shB7-H3 cells are lower than those in the Caco-2-NC cells after B7-H3 was knocked down (Figure 6D). **E.** Western blotting was used to detect the Smad1, Smad9, pSmad1/5/9 levels in the SW480-NC, SW480-B7-H3, SW480-B7-H3+AG490 (a specific inhibitor of Jak2), SW480-B7-H3+LY294002 (a specific inhibitor of PI3K), SW480-B7-H3+AG490+LY294002, Caco-2-NC, and Caco-2-shB7-H3 cells. The data shows that the SW480-B7-H3 cells express more Smad1, Smad9 and pSmad1/5/9 than the SW480-NC cells, while the expression levels of Smad1, Smad9 and pSmad1/5/9 were decreased in the Caco-2 cells after B7-H3 was knocked down (Caco-2-shB7-H3 cells compared with Caco-2-NC cells). The elevated expression of Smad1 and Smad9 in the SW480-B7-H3 cells was not significantly inhibited by AG490. Meanwhile, it was significantly inhibited by LY294002 and both AG490 and LY294002, indicating that PI3k was the major signaling pathway by which B7-H3 up-regluated Smad1 and Smad9. **F.** The Akt expression levels were not markedly different in any of the cells: SW480-NC, SW480-B7-H3, SW480-B7-H3+Ly294002, Caco-2-NC and Caco-2-shB7-H3. Nevertheless, the pAkt levels were noticeably elevated after the SW480 cells were transfected with B7-H3 (SW480-B7-H3 cells compared with SW480-NC cells), and observably decreased when B7-H3 was knocked down (Caco-2-NC cells compared with Caco2-shB7-H3 cells). B7-H3 decreased Smad1 and pAkt expression, which were blocked by Ly294002. (SW480-B7-H3 cells compared with SW480-B7-H3+Ly294002 cells). **H.** Smad1 was inhibited by siRNAs with differece sequences, siRNA1 and siRNA2, and an siRNA with a nonsense sequence was used as an internal control. Smad1, pSmad1, N-cadherin, Vimentin, and β-catenin were analyzed. **G.**, **I.** The density of each band in F and **H.** was obtained with ImageJ 1.48. Differences were considered to be statistically significant when the *p* values were <0.05. and then increased the expression of SMAD1 to promote the EMT in cancer cells.

To deeply investigate the mechanism by which B7-H3 induces the EMT in colorecal cancer cells, we used an Agilent transcript chip to scan the genes that were likely involved in this process. First, we found that the mRNAs for 2,813 genes were upregulated in the SW480-B7-H3 cells compared with the SW480-NC cells, and 1,133 genes were down-regulated after B7-H3 was knocked down in the Caco-2 cells. The intersection of the differentially expressed genes in these two assemblages includes 227 genes (Figure 6A).

Then, we performed gene ontology (GO) and KEGG Pathway enrichment analyses to cluster these 227 differentially expressed genes. The results of the KEGG Pathway enrichment analysis showed that these differentially expressed genes could be classified into the T cell receptor signaling pathway, Melanogenic pathway, TGF-β signaling pathway and other immunity or metabolism-related pathways (Figure 6B). Interestingly, the TGF-β signaling pathway is known to be a classic signaling pathway that induces the EMT in cancer cells. Moreover, Smad1 and Smad9 (sometimes referred to as Smad8) mediate BMP (Bone morphogenetic proteins) signaling and bind to a Co-Smad (Smad4) to form R-Smad/Co-Smad complex, which is then translocated to the nucleus to act as a transcription factor that induces the expression of downstream genes and causes the EMT in the cell.

We used real-time PCR and western blotting to double validate the results of the transcript chip to ensure that B7-H3 upregulated the expression of Smad1 in both the B7-H3-overexpressing SW480-B7-H3 cells and Caco-2-NC cells. As shown in Figure 6C and 6D, the SW480-B7-H3 cells expressed higher levels of B7-H3 than the control SW480-NC cells, and Smad1 and Smad9 were also expressed at higher levels compared with the SW480-NC cells. As the B7-H3 knockdown model, the Caco-2-shB7-H3 cells, which expressed lower levels of B7-H3 than the Caco-2-NC cells, expressed lower levels of Smad1 and Smad9. These results were also confirmed in the western blotting experiments (Figure 6E).

Jak2-STAT3 and PI3K-Akt are reported to be the downstream signaling pathways that mediate the ability of B7-H3 to regulate the expression of Bcl-2, Bcl-xl, Bax and other molecules. In a subsequent study, we also added AG490 (a specific inhibitor of Jak2, 100 μM), LY294002 (a specific inhibitor of PI3K, 100 μM), or both AG490 and LY294002 to the B7-H3-overexpressing SW480-B7-H3 cancer cells. Figure 6E showed that SW480-B7-H3 expressed significantly higher levels of Smad1 and Smad9 than the SW480-NC cells. After the addition of LY294002 or both LY294002 and AG490, the expression of Smad1 and Smad9 decreased in these samples, whereas in the samples that were only treated with AG490, the expression of Smad1 and Smad9 was not significantly decreased. Therefore, we inferred that PI3K-Akt might be a major pathway by which B7-H3 regulated the expression of Smad1 and Smad9.

To confirm these results, we repeated the same experiment in SW480-NC and SW480-B7-H3 cells that were treated with or without LY294002 and also analyzed the levels of phosphorylated Akt by western blot. The level of phosphorylated Akt was significantly increased in the SW480-B7-H3 cells compared with the SW480-NC cells, and the expression of Smad1 also increased. When the SW480-B7-H3 cells were treated with LY294002, the B7-H3-induced increases in Akt phosphorylation and Smad1 expression were blocked by LY294002. These results also proved that the PI3K-Akt pathway was the major signal transduction pathway by which B7-H3 regulated Smad1.

To prove that Smad1 was involved in the B7-H3-induced EMT in the colon cancer cell lines, we used two different Smad1-specific small interfering RNAs with different sequences to inhibit the expression of Smad1, and then analyzed N-Cadherin and Vimentin expression, which indicated that the cells had a mesenchymal phenotype. As shown in Figure 6H and 6I, the expression of N-Cadherin and Vimentin was significantly decreased in the SW480-B7-H3 co-incubated with Smad1 siRNA-1 or Smad1 siRNA-2 compared with the SW480-B7-H3 cells that had been co-incubated with siRNA-NC (negative control). These interference experiments indicated that the Smad1 siRNAs could effectively inhibit the function of B7-H3 in the EMT.

### B7-H3 expression increased the number of colon cancer cells with the and promoted xenograft growth

We also established two pairs of colon cancer xenografts for *in vivo* studies. SW480-NC & SW480-B7-H3 cells and Caco-2-NC & Caco-2-shB7-H3 cells were injected into the axilla of nude mice. The sizes of the xenografts were measured twice every week. After approximately 30 days, the tumor volumes were different in both the SW480 pairs and Caco-2 pairs. The differences between the xenografts sizes with higher B7-H3 expression and lower expression were increased with time. In 8 weeks, the mean volumes of the xenografts in the SW480-B7-H3 group were significantly larger than the SW480-NC group (*p* < 0.05), and the mean volumes of the xenografts in the Caco-2-NC group were also significantly larger than the Caco-2-shB7-H3 group, in which B7-H3 expression in the Caco-2 cells has been knocked down.

**Figure 7:**
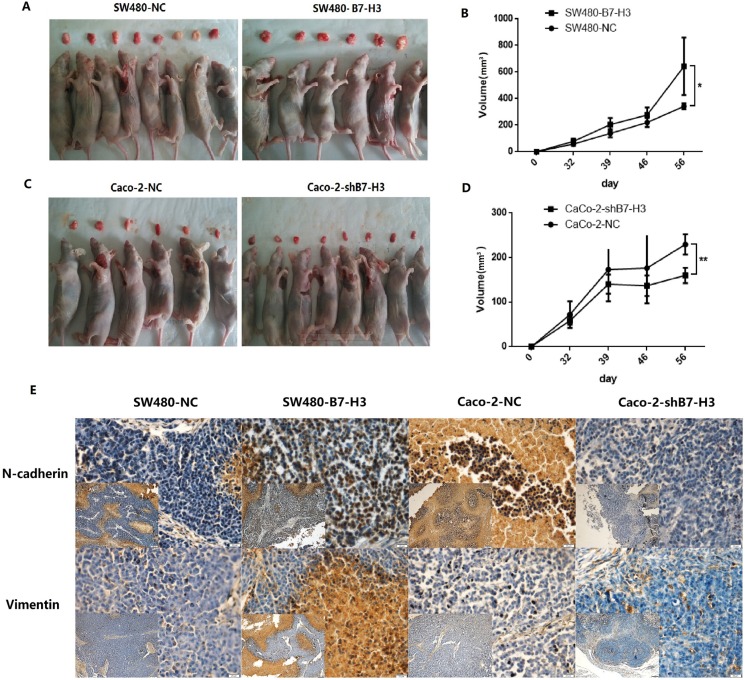
B7-H3 expression increased the mesenchymal phenotype of colon cancer cells and promoted xenograft growth **A.**, **B.** Approximately 2.0×10^6^ SW480-NC or SW480-B7-H3 cells were subcutaneously injected into the lower flanks of the mice. The widths and lengths of the xenografts were examined each week. SW480-B7-H3 cells, which expressed a high level of B7-H3, promoted tumor growth compared with the SW480-NC cells (*p* < 0.01). **C.**, **D.** Approximately 2.0×10^6^ Caco-2-NC or Caco-2-shB7-H3 cells were subcutaneously injected into the lower flanks of the mice. The widths and lengths of the xenografts were examined each week. Caco-2-NC cells caused rapid growth of the xenografts compared with the Caco-2-shB7-H3 cells in which B7-H3 expression has been knocked down (*p* < 0.05). **E.** Immunohistochemical staining of the xenografts. N-cadherin and Vimentin antibodies were used to stain tissue slices of the mouse xenografts formed by the SW480-NC, SW480-B7-H3 and Caco-2-NC, and Caco-2-shB7-H3 cells to show the alterations in the mesenchymal phenotype. The magnifications are 100× (larger picture) or 400× (smaller picture). The data showed that the SW480-B7-H3 cells has express more N-cadherin and Vimentin than the SW480-NC cells, and the Caco-2-NC also express more N-cadherin and Vimentin than the Caco-2-shB7-H3 cells in which B7-H3 has been knocked down.

To determine the function of B7-H3 in the EMT *in vivo*, we collected and fixed the xenografts in formaldehyde. The N-cadherin and Vimentin expression levels in the xenografts were examined by immunohistochemistry. Figure 7C shows that the tumors that were which formed by the SW480-B7-H3 cells also displayed higher N-cadherin and Vimentin expression *in vivo* compared with the tumors formed by the SW480-NC cells. In contrast, when B7-H3 was knocked down in Caco-2 cells, the tumor displayed lower N-cadherin and Vimentin expression levels.

## DISCUSSION

Our data showed, for the first time, that B7-H3 promoted the EMT in colorectal cancer cells. B7-H3 overexpression could active the PI3K-Akt pathway to increase the expression of Smad1, a key molecule in the TGF-β signaling pathway.

We also proved that B7-H3 was co-expressed with MMP2 and MMP9 in colorectal cancer patients. These results may explain why abnormal B7-H3 association was associated with a poor overall survival in colorectal cancer patients, which had also been observed in several other cancers. In subsequent studies, we observed that B7-H3 increased the invasion and migration of colorectal cancer cells *in vitro*. The transformation of the B7-H3-overexpressing SW480 cells from a cobblestone-like morphology to a long spindle-like morphology and other evidence indicated that B7-H3 induced the EMT in cancer cells. Both the WB and immunofluorescence experiments proved that E-Cadherin and β-catenin expression were downregulated by B7-H3 overexpression, while N-Cadherin and Vimentin expression were up-regulated by B7-H3 overexpression.

B7-H3 was first recognized as a costimulatory molecule for T cell activation, proliferation and IFN-gamma production in 2001 [17]. In 2003, Ling V revealed that B7-H3 inhibited CD4^+^ T cell proliferation and downregulated cytokine production [30]. Steinberger P supported the opposite hypothesis in 2004 [31]. Therefore, the exact function of B7-H3 in regulating T cell and cytokine production is still being debated. In contrast, the relationship between increased B7-H3 expression and poor overall suvival has been confirmed in many cancer types, including colorectal cancer [32], leukemia [33], and non-small lung cancer [34]. Interestingly, Ingebrigtsen showed that the nuclear localization of B7-H3 was an independent risk factor for a poor overall survival [35]. Our team demonstrated that B7-H3 could active the Jak2-STAT3 signaling pathway to up-regulate the expression of the anti-apoptotic Bcl-2 family members Bcl-2 and Bcl-xl and down-regulate the expression of the pro-apoptotic protein Bax to inhibit cancer cell apoptosis and cause drug resistance in cancer cells [28].

B7-H3 enhanced the inflammatory response and promoted MMP-9 expression in a pneumococcal meningitis animal model [36]. In osteosarcoma, B7-H3 promoted cell invasion and upregulated matrix metalloproteinase 2 expression (MMP-2) [37]. Downregulation of B7-H3 with an siRNA reduced melanoma [38] and breast cancer cell migration and Matrigel invasion [39]. In our study, we revealed that both MMP-9 and MMP-2 were co-expressed with B7-H3 in colorectal cancer tissue; the correlation coefficients were 0.7492 and 0.5643, respectively. The results indicated that B7-H3 had a close relationship with MMP9 and MMP2 expression in cancer cells. *In vitro*, the SW480-B7-H3 cells acquired more invasion and migratory capabilities than their counterpart SW480-NC cells, whereas the invasion and migration of the Caco-2-shB7-H3 cells were inhibited. In both the *in vivo* and *in vitro* experiments, signal transducer and activator of transcription 3 (STAT3) phosphorylation was decreased in the B7-H3 knockdown cell variants [26, 40], whereas STAT3 phosphorylation was increased in the B7-H3-over-expressing cancer cells [28]. Jak2-STAT3 activity has been shown to promote MMP expression in several studies [41-43]. Therefore, B7-H3 might regulate MMP expression, cell migration, and invasion *via* the Jak2-STAT3 signaling pathway, and we confirmed this hypothesis in cell-based experiments [44].

The EMT is a critical initiating event in the metastatic cascade of colorectal cancer [29]; therefore, we hypothesized that B7-H3 caused the EMT in cancer cells and that B7-H3 promoted MMPs expressions as one characteristic of the EMT. According to western blot results, the SW480 cells lost the expression of epithelial markers (E-Cadherin and β-catenin) and acquired mesenchymal features (N-Cadherin and Vimentin) after transfection with B7-H3, whereas the Caco-2 cells expressed higher levels of E-Cadherin after B7-H3 was down-regulated. The expression levels of B7-H3, E-Cadherin, N-Cadherin, Vimentin and β-catenin were also evaluated by immunofluorescence. These results indicated that the cells had undergone an EMT process as a result of the increased expression of B7-H3.

As suggested by their name, tumor-initiating cells (TIC) [45] are a rare subpopulation of cells that can retain self-renewal capacity and generate xenografts or metastases in distant organs. CD133, CD44 are known as bio-marker of colorectal TIC [46]; Oct4 expression is associated with early stages of stem cell development [47]. Bin Z and colleagues had reported that B7-H3 was co-expressed with CD133 in colorectal cancer tissue samples and was associated with cancer metastasis and poor progression [48]. In our study, we proved that B7-H3 over-expression in SW480 cells could increase the expression of CD133. Moreover, we also certified that CD133 expression could be reduced after B7-H3 expression was inhibited by siRNAs in Caco-2 cells. In addition to CD133, abnormal B7-H3 expression also increased CD44 and Oct4 expression. In summary, B7-H3 induced cancer cells to express more stemness biomarkers, indicating that the cancer cells exhibited greater self-renewal, distal metastasis capabilities and drug resistance. Because stronger stemness is another property of the EMT, the hypothesis is also supported these results of B7-H3 in TIC.

According to recent reports [49], Smad9 acts as an inhibitory Smad (I-Smad) and reduces BMP activity. Thus, we focused our subsequent studies on Smad1. Jak2-STAT3 has been reported to be involved in the B7-H3 bio-regulation process. The mechanism by which B7-H3 activates PI3K-Akt was only mentioned in Cathrine Pedersen's doctoral dissertation. The PI3K-Akt pathway has been reported to increase the expression of Smad1 [50]. Therefore, we investigated the relative levels of proteins in both the Jak2-STAT3 and PI3K-Akt pathways and Smad1 by treating the SW480-B7-H3 cells with AG490, a Jak2-specific inhibitor, and LY294002, a PI3K-specific inhibitor. Based on the western blot results, AG490 did not inhibit the function of B7-H3 in upregulating Smad1 expression, and LY294002 could inhibit most of the effects of B7-H3. In subsequent studies, we proved that LY294002 successfully inhibited Akt phosphorylation in the cells that expressed higher levels of B7-H3 and could also suppress the expression of Smad1.

Small interfering RNAs were used to inhibit the expression of Smad1. In the SW480-B7-H3 cells, siRNAs targeting Smad1 could successfully block the function of B7-H3 in inducing the EMT. These results also proved that Smad1 played a key role in the mechanism by which B7-H3 promoted the EMT.

*In vivo* studies, We also proved that B7-H3 up-regulated N-cadherin and Vimentin expression. These results were similar to the B7-H3-induced alterations in N-cadherin and vimentin expression *in vitro*. Both of these results implied that B7-H3 increased the number of colon cancer cells with the mesenchymal phenotype *in vitro* and *in vivo*.

Therefore, our data revealed that B7-H3 could promote cancer cell transformation into a more mesenchymal phenotype with cancer stem cell characteristics. These changes helped the cancer cells become isolated from the cancer tissue and decrease their adhesion to the adjacent cells and cell matrix. More stem cell biomarkers were detected in the cancer cells that over-expressed B7-H3, implying that these transformed cells had a greater self-renewal ability and drug resistance capacity and could survive longer in the circulatory system. B7-H3 could increase the expression of Smad1, a molecule in the TGF-β pathway, by activating the PI3K-Akt pathway to promote the EMT.

Finally, our investigation proved that B7-H3 induced the EMT in cancer cells. B7-H3 had a close relationship with the T stage of cancer and was co-expressed with MMP2 and MMP9 in cancer tissues, which might originate from its ability to promote the EMT.

## MATERIALS AND METHODS

### Antibodies and reagents

The anti-human B7-H3, MMP2 and MMP9 antibodies used for immunohistochemistry were purchased from Santa Cruz Biotechnology, Inc. (Dallas, TX, United States). The anti-human B7-H3, E-Cadherin, N-Cadherin, Vimentin, β-catenin, CD44, Oct4, Akt, phosphorylated Akt, Smad1 and Smad9 antibodies were from Abcam (Cambridge, MA, United States). The anti-human phosphorylated Smad1/5 and Smad1/5/9 antibodies were from Cell Signaling Technology, Inc. (Danvers, MA, United States). The anti-human CD133 antibody for flow cytometry was purchased from Miltenyi Biotec (Bergisch Gladbach, Germany). The horseradish peroxidase-conjugated secondary anti-mouse and anti-rabbit antibodies and the GAPDH antibody were from Beyotime (Nantong, China). The fluorescent dye DiI that was used to label the cell membrane was from Keygentec (Nanjing, China). The Jak2-specific inhibitor AG490 was purchased from Sigma-Aldrich (St. Louis, MO, United States). The PI3K-specific inhibitor LY294002 was from Selleckchem (Houston, TX, United States). The Matrigel^TM^ matrix used in the invasion assay was from BD (San Jose, CA, United States).

### Patient tissues and cell lines

In total, 87 patients with CRC were enrolled from the affiliated hospital of Jiangnan University, China between July 2008 and December 2009. Fresh tissues were harvested from the patients, snap-frozen, and preserved at −80°C. The clinical characteristics of the colorectal cancer patients were included in Table 1. The SW480 and CaCo-2 human colorectal cancer cell lines were maintained in our lab. The SW480 cells were cultured in RPMI 1640 medium. The CaCo-2 cells were maintained in MEM. We constructed SW480 cells that expressed high levels of B7-H3 (SW480-B7-H3) and CaCo-2 cells that were stably transfected with a B7-H3 shRNA (CaCo-2-shB7-H3). Cells that had been transfected with a mock vector were used as negative controls (SW480-NC and CaCo-2-NC). All of the media (HyClone, GE Healthcare Life Sciences, South Logan, UT, United States) were supplemented with 10% fetal bovine serum (Clark Bioscience, Houston, TX, United States). The cells were incubated at 37°C in a humidified atmosphere with 5% CO_2_.

### Immunohistochemistry

The colorectal cancer samples were fixed and embedded in paraffin. The sections (5 μm) were routinely processed and stained with an appropriate concentration of a primary monoclonal antibody overnight, followed by incubation with a horseradish peroxidase-conjugated secondary antibody (GTVisionII Immunohistochemistry Detection Kit for Rabbit/Mouse, Gene Tech, Shanghai, China). The positively stained areas throughout the tissue section were graded according to the percentage of positively stained cells: 0 for < 25%; 1 for 25-49%; 2 for 50-74%; and 3 for 75-100%. In this study, 0 was considered negative or moderate staining, while 1, 2 and 3 were considered positive staining.

### Western blotting

Western blotting was performed on whole-cell extracts prepared by lysing 1 × 10^6^ cells in RIPA lysis buffer containing phosphatase inhibitors, protease inhibitors and 100 mmol/L PMSF (KeyGEN BioTECH, China) for 20 min on ice. The proteins were separated on 10% SDS-PAGE gels and then transferred onto a PVDF membrane (Merck Millipore, Germany). The membranes were blocked with 5% nonfat dry milk for 1 h at room temperature and then incubated with a 1:1,000 dilution of the indicated antibodies overnight at 4°C, followed by incubation with a secondary antibody for 1 h at room temperature. The immunoreactive bands were visualized using Beyo ECL Plus (Beyotime, China).

### Cellular morphology assay

The cells were plated in a confocal dish until they reached 60%-70% confluence. A 0.5% DiI solution dissolved in fresh media was added to the dish and incubated at 37°C for 30 min; then, the cells were washed 3 times with PBS. Next, the cells were fixed with 4% paraformaldehyde for 30 min at RT and washed three times with PBS. The cell nuclei were stained with DAPI for 10 min at RT in the dark, and washed 3 more times with PBS. Finally, a TSC SP8 laser confocal scanning microscope (Leica, Germany) was used to capture the images and record the cellular morphology.

### Immunofluorescence

First, we grew 1.5×10^5^ cells on coverslips in a 24-well plate in culture media. Once the cells had adhered and reached 50% confluence, the media was aspirated from the plates, and the cells were washed twice with PBS. The cells were fixed with 4% paraformaldehyde for 20 min at 4°C and washed three times with PBS. Then, 0.2% TritonX-100 was incubated with cells for 20 min at room temperature (RT), and the cells were washed 3 times with PBS. The cells were blocked with 1% BSA and 4% goat serum solubilized in PBS for 30 min at RT and washed once with PBS. According to the manufacturer's instructions, we used different dilutions of the primary antibodies to stain the cells overnight at 4°C. Then, the cells were washed 3 times with PBS for 5 min. Subsequently, we incubated the cells with a PE-conjugated secondary antibody for 1 h at RT, and then washed them 3 times with PBS. The cell nuclei were stained with DAPI for 10 min at RT, and washed again 3 times with PBS. Finally, the coverslip was removed and mounted on a slide with anti-fade agent. An IX71 inverted fluorescence microscope (Olympus, Japan) was used to observe the expression of the surface markers.

### Flow cytometry

We diluted the cells to 5×10^6^ cells/ml with PBS and aliquoted 100 μL of cells per tube. Ten microliters of appropriately diluted primary antibodies were added to each tube. The cells were incubated for 20 min at 4°C in the dark and washed once with PBS. Then, the supernatant was discarded, and the cells were resuspended by gently flicking the tube. Next, 500 μL PBS were added to each tube prior to analysis. If the primary antibody was not directly coupled, we did not add PBS. Instead, 10 μL of an appropriate secondary antibody was added and incubated for 20 min at 4°C in the dark. The cells were washed with PBS, the supernatant was discarded, and the cells were resuspended. Finally, 500 μL of PBS was added to each tube, and the cells were analyzed with a FACSCantoII flow cytometer (BD, Franklin Lakes, NJ, United States).

### Invasion and migration assays

On the first day, we diluted Matrigel (Corning, Tewksbury, MA, United States) in cold serum-free cell culture media at a 1:8 dilution. Then, 100 μL of the diluted Matrigel was added to the upper chamber of the Transwells (Corning, Tewksbury, MA, United States) in a 24-well plate. The Transwell was incubated at 37°C overnight. Meanwhile, the cells were also starved in serum-free media overnight. On the next day, the cells were harvested and resuspended in media containing 1% FBS at a density of 2×10^5^ cells/ml. Then, the gelled Matrigel was gently washed with warm serum free-culture media. Next, 100 μL of the cell suspension was plated on the Matrigel. The lower chamber of the Transwell was filled with 600 μL of culture media containing 5 μg/mL fibronectin as an adhesive substrate. The 24-well plate was incubated at 37°C for 24 h. For the migration assay, we directly plated cells into the Transwell chambers, and incubated them at 37°C for 48 h. The Transwells were removed from the 24-well plates, and the non-migrated cells on the top of the Transwell were scraped off. The migrated cells were fixed with 4% paraformaldehyde and stained with a 0.01% crystal violet solution. The images were collected using a fluorescence microscope (IX71).

### Wound healing assay

The cells were cultured to confluence in a 6-well plate, and the wounds were scratched using a sterile 20 μL pipet tip. The cells were gently rinsed with PBS, and 2 mL of media containing 0.5% serum was added. We captured images every 24 h to record the gaps in the cell monolayers using an inverse microscope (IX71).

### RNA isolation, purification and first strand cDNA synthesis

The total RNA was isolated from 1.5 × 10^6^ cells using TRIzol, according to the manufacturer's instructions, and quantified with a NanoDrop 2000 (Thermo Scientific, Waltham, MA, United States). The total RNA was treated with RNase-free DNase to remove the residual genomic DNA. The first strand cDNAs were synthesized from 1 μg of RNA using oligo-dT primers and AMV reverse transcriptase.

### Relative real-time polymerase chain reaction

The expression levels of the B7-H3, Smad1 and Smad9 transcripts were analyzed relative to the level of the transcript of the β-actin gene using a ViiA^TM^ 7 Real-Time PCR System (Applied Biosystems Inc., Foster City, CA, United States). The first-strand cDNAs were amplified in a 20 μL PCR reaction mixture containing 10 μL of 2 × SYBR green PCR master mix, 0.4 μL of 50 × ROX, 0.4 μL of each specific primer set, and ddH_2_O to a volume of 20 μL. The sequences of primers were as follows: β-actin 5′- AGCGAGCATCCCCCAAAGTT-3′ (sense), 5′- GGGCACGAAGGCTCATCATT-3′ (antisense); B7-H3 5′-AGCACTGTGGTTCTGCCTCACA-3′ (sense), 5′-CACCAGCTGTTTGGTATCTGTCAG-3′ (antisense); Smad1 5′- CTCTCAGCCGATGGACACAA -3′ (sense), 5′- TTGTGGAGGAGGCATGGAAC -3′ (antisense); and Smad9 5′- ATGTGATTTACTGTCGCGTGT -3′ (sense), 5′- CGGTAGTGGTAAGGGTTAATGC -3′ (antisense). The PCR cycles consisted of 40 cycles of amplification of the cDNAs with annealing at 55°C, except for B7-H3, which was annealed at 60°C.

### RNA interference

The human Smad1 siRNA was synthesized by Ribobio (Guangzhou, China). The SW480-B7-H3 cells were transfected with 150 nM Smad1 siRNA-1 (GAGCCACCATGAACTAAAA), Smad1 siRNA-2 (GCATCAATCCCTACCACTA) or the negative control siRNA using the transfection reagent Lipofectamine 2000 (Invitrogen, New York, NY, United States). The cells were incubated for 48 h after transfection and then used for western blotting.

### RNA extraction, labeling and hybridization

RNA extraction and purification have been described previously. The Agilent human 8×60 transcription microarray chips (Agilent Technologies, Santa Clara, CA, United States) were employed in this study. The cDNAs were labeled with a fluorescent dye (Cy5 and Cy3-dCTP) using Eberwine's linear RNA amplification method and a subsequent enzymatic reaction. Double-stranded cDNAs (containing the T7 RNA polymerase promoter sequence) were synthesized from 100 ng of total RNA using the CbcScript reverse transcriptase from the cDNA synthesis system and the T7 Oligo (dT), according to the manufacturer's protocol (Capitalbio, Beijing, China).

The dsDNA products were purified using a PCR NucleoSpin^®^ Extract II Kit (Macherey-Nagel, Duren, Germany) and eluted with 30 μL of elution buffer. The eluted double-stranded cDNA products were evaporated under a vacuum to 16 μL and included in the 40 μL *in vitro* transcription reactions that were incubated with a T7 Enzyme Mix for 14 h at 37°C. The amplified cRNA was purified using the NucleoSpin^®^ RNA Clean-up Kit (Macherey-Nagel, Duren, Germany).

The hybridization solution contained 3×SSC, 0.2% SDS, 5×Denhardt's solution and 25% formamide. DNA in hybridization solution was denatured at 95°C for 3 min prior to loading onto a microarray. The arrays were hybridized in an Agilent Hybridization Oven overnight at 42°C and a rotation speed of 20 rpm and then washed with two consecutive solutions (0.2% SDS, 2×SSC at 42°C for 5 min, and 0.2×SSC for 5 min at room temperature).

### Microarray imaging and data analysis

The array data were analyzed for data summarization, normalization and quality control using the GeneSpring software V12 (Agilent). To select the differentially expressed genes, we used threshold values of ≥2 and ≤-2-fold change.

### *In vivo* studies

Athymic BALB/c nude mice (4 weeks old, female, SPF grade) were obtained from the Shanghai Animal Research Center and housed in a certified vivarium facility at constant temperature (23±2°C) and humidity (50-70%) with a 12 h light-dark cycle. Approximately 2.0×10^6^ SW480-NC or SW480-B7-H3 cells were subcutaneously inoculated into the lower flanks of the mice. The same number of Caco-2-NC or Caco-2-shB7-H3 cells was also used to inject the lower flanks of the mice. The xenograft volumes were calculated according to the following formula: volume = (width)^2^×length/2. The growth curve of each tumor was plotted. After 2 months, the xenografts were isolated from the animal model and fixed in 10 % phosphate-buffered formaldehyde at room temperature for 48 h. The N-cadherin and Vimentin expression levels in the xenografts were examined by immunohistochemistry of tissue slices.

### Statistical analysis

Differences in the mean values between groups were analyzed by a non-paired t-test. At least three independent experiments were performed for all studies. Differences were considered to be statistically significant when the *p* values were < 0.05. All data were analyzed using GraphPad Prism 6 (GraphPad Software Inc., La Jolla, CA, United States).
